# Krüppel-like factor 6 (KLF6) promotes cell proliferation in skeletal myoblasts in response to TGFβ/Smad3 signaling

**DOI:** 10.1186/2044-5040-3-7

**Published:** 2013-04-02

**Authors:** Mathew G Dionyssiou, Jahan Salma, Mariya Bevzyuk, Stephanie Wales, Lusine Zakharyan, John C McDermott

**Affiliations:** 1 Department of Biology, York University; York University, 4700 Keele St, Toronto, ON, M3J 1P3, Canada; 2 Centre for Research in Mass Spectrometry, York University; York University, Toronto, ON, M3J 1P3, Canada; 3 Muscle Health Research Centre, York University; York University, Toronto, ON, M3J 1P3, Canada; 4 Centre for Research in Biomolecular Interactions, York University; York University, Toronto, ON, M3J 1P3, Canada

**Keywords:** Myoblasts, Krüppel-like factor 6, Transforming growth factor β, Cell proliferation

## Abstract

**Background:**

Krüppel-like factor 6 (KLF6) has been recently identified as a MEF2D target gene involved in neuronal cell survival. In addition, KLF6 and TGFβ have been shown to regulate each other’s expression in non-myogenic cell types. Since MEF2D and TGFβ also fulfill crucial roles in skeletal myogenesis, we wanted to identify whether KLF6 functions in a myogenic context.

**Methods:**

KLF6 protein expression levels and promoter activity were analyzed using standard cellular and molecular techniques in cell culture.

**Results:**

We found that KLF6 and MEF2D are co-localized in the nuclei of mononucleated but not multinucleated myogenic cells and, that the MEF2 *cis* element is a key component of the KLF6 promoter region. In addition, TGFβ potently enhanced KLF6 protein levels and this effect was repressed by pharmacological inhibition of Smad3. Interestingly, pharmacological inhibition of MEK/ERK (1/2) signaling resulted in re-activation of the differentiation program in myoblasts treated with TGFβ, which is ordinarily repressed by TGFβ treatment. Conversely, MEK/ERK (1/2) inhibition had no effect on TGFβ-induced KLF6 expression whereas Smad3 inhibition negated this effect, together supporting the existence of two separable arms of TGFβ signaling in myogenic cells. Loss of function analysis using siRNA-mediated KLF6 depletion resulted in enhanced myogenic differentiation whereas TGFβ stimulation of myoblast proliferation was reduced in KLF6 depleted cells.

**Conclusions:**

Collectively these data implicate KLF6 in myoblast proliferation and survival in response to TGFβ with consequences for our understanding of muscle development and a variety of muscle pathologies.

## Background

KLF6 is a member of the Krüppel-like Factors (KLF) gene family which are a group of transcription factors that contain three highly conserved Cys_2_-His_2_ type zinc fingers located in the C-terminus [1,2]. Subsequently, these proteins regulate a vast range of target genes by preferentially binding to cognate GC-boxes or CACCC elements. KLF6 was originally identified due to its ability to regulate TATA-less gene promoters that can regulate glycoproteins in placental cells [3]. Since then, KLF6 has been found to be expressed in most tissues including neuronal, hindgut, heart and limb buds [4] and is localized in the nucleus [5]. Interestingly, homozygous null *KLF6* mice result in failure in the development of the liver and yolk sac vasculature, resulting in early lethality at (E)12.5 [4]. To date, the most well-established target gene of KLF6 is Transforming growth factor β (TGFβ) and its receptors [6], and subsequent studies have shown a positive feedback loop by which TGFβ activation enhances KLF6 transactivation properties through the formation of a Smad3-Sp1-KLF6 protein complex [7]. TGFβ and KLF6 cooperatively regulate a wide range of cellular processes such as cell differentiation, proliferation and epithelial-to-mesenchymal transitions (EMT) [8-13]. Recently KLF6 was identified as a myocyte enhancer factor 2 (MEF2) target gene that is involved in neuronal cell survival [14]. Since TGFβ and MEF2 are two key regulators of skeletal myogenesis and since KLF6 was identified in the myogenic transcriptome [15], we wanted to investigate the role of KLF6 in skeletal muscle cells.

Regulation of skeletal myogenesis is a complex process. Initially paracrine factors instigate the migration of designated myotome progenitor cells to the dermomyotome region of the somite. These proliferating cells grow and divide until cell contact triggers differential gene expression and activation of the MEF2 proteins and muscle regulatory factors (MRFs). This cascade of events causes morphological changes in the progenitor cells that allow them to align and fuse to form multinucleated myotubes that can eventually spontaneously contract as functional muscle fibers. TGFβ antagonizes this process by preventing cells from exiting the cell cycle hence maintaining myoblasts in a proliferative state. TGFβ ligands bind to a type II receptor which becomes activated and autophosphorylated [16]. The activated type II receptor can then phosphorylate and activate a type I receptor, which in turn phosphorylates receptor-mediated Smads(2/3) enabling them to dimerize with Smad4 and translocate into the nucleus where they can bind to other transcription factors and DNA, to repress essential muscle genes and the expression of their downstream targets [17,18]. In addition, TGFβ also regulates the mitogen-activated protein kinase (MAPK) pathway, which involves a cascade of protein kinases (MAPKKK, MAPKK, MAPK) that become activated in sequence by G-proteins in response to TGFβ binding its receptors [19-21]. Upon TGFβ activation, MEK1/2 (MAPKK) can phosphorylate and activate Extracellular signal-regulated kinase (ERK)1/2 MAPK at conserved TEY sites, causing it to translocate into the nucleus to regulate gene expression. These two TGFβ-regulated pathways converge to inhibit the function of MEF2 and hence muscle-specific genes [22], and ultimately result in cell proliferation [23,24]. Not surprisingly, inhibition of either or both of these pathways, (either pharmacologically or through ectopically expressed Smad7, which can antagonize the canonical Smad-pathway), enhances myotube formation [25,26]. Crosstalk between these pathways is further supported by Smad7 antagonizing the repressive effects of MEK1 on MyoD [26,27].

In this report, our goal was to assess the role of KLF6 in myogenic cells based on its regulation by both MEF2D and TGFβ. We report that TGFβ upregulates KLF6 specifically through a Smad3-dependent pathway, which enhances proliferation in myoblasts. In addition, we observed that 1) TGFβ enhanced KLF6 promoter activation, and 2) that MEF2 is recruited to the KLF6 promoter region but is not required for KLF6 activation by TGFβ. Pharmacological inhibition of Smad3 repressed KLF6 expression by TGFβ and cell proliferation but, importantly did not re-activate the differentiation program which is potently repressed by TGFβ signaling. Conversely, TGFβ treatment coupled with pharmacological inhibition of MEK1/2, enhanced myotube formation but had no effect on KLF6 expression and function. Loss of function assays using siRNA targeting KLF6 revealed that KLF6 is required for cell proliferation. These experiments tease apart two independent functions of TGFβ signaling in myogenic cells. One is a repressive effect on differentiation which is mediated by ERK activation, the other being an enhancement of proliferation, which is dependent on Smad3 and KLF6.

## Methods

### Plasmids

Expression plasmids for pcDNA3-MEF2D, pCMV β-galactosidase [28,29], and reporter gene constructs for 3TP-lux [30], MCK-Luc [31], and MEF2-Luc [32] have been previously described. KLF6 reporter constructs pRMO6 and pROM6 ΔMEF2 were generously provided by Dr. Nicolas P. Koritschoner (Faculty of Bioquimica y Ciencias Biologicas, Universidad Nacional del Litoral, Santa Fe, Argentina).

### Antibodies

Anti-MEF2A rabbit polyclonal, anti-Myosin heavy chain mouse monoclonal and anti-Myogenin mouse monoclonal antibodies were produced with the assistance of the York University (Toronto, Ontario, Canada) Animal Care Facility. Anti-MEF2D (1:1000; BD Biosciences, Mississauga, Ontario, Canada), Smad3, phospho-Smad3 and phospho-ERK1/2 (1:1000; Cell Signaling, Toronto, Ontario, Canada), and KLF6, actin, and ERK1/2 (1:1000; Santa Cruz, Santa Cruz, CA95060, US) were used for immunoblotting experiments. Immunoglobulin G (IgG) was also purchased from Santa Cruz Biotechnologies.

### Cell culture, transfections and drug treatments

C2C12 cells were maintained in DMEM supplemented with 10% fetal bovine serum (HyClone, Rockford, IL61101, US), 1% L-glutamine and 1% penicillin-streptomycin. Cells were maintained in a humidified, 37°C incubator with a 5% CO_2_ atmosphere. For transfections, cells were seeded on pre-gelatin-coated plates 1 day prior to transfection and were transfected according to the standard calcium phosphate method previously described by Perry *et al.*, 2001. A mixture of 50 μl 2.5 M CaCl_2_ per 25 μg DNA with an equal volume of 2× HeBS (2.8 M NaCl, 15 mM Na_2_HPO_4_, 50 mM 4-(2-hydroxyethyl)-1-piperazineethanesulfonic acid (HEPES), pH = 7.15) was used, and the cells were incubated overnight followed by washing and addition of fresh media. Drug treatments were used at the following concentrations: 2 ng/ml TGFβ, 5 μM Sis3 and 10 μM U0126 as indicated.

### siRNA gene silencing

siRNA targeting KLF6, MEF2D and non-specific scramble RNA were purchased from Sigma. Transient transfections were performed using TurboFect Transfection Reagent (R0531, Fermentas) according to the manufacturer’s instructions. Turbofect (Fermentas): a 1:2 mixture ratio of DNA to turbofect reagent (including 4 ng/ml siRNA) in 200 μl serum-free DMEM was prepared for 19-h incubation.

### Immunocytochemistry

C2C12 cells were treated as previously described by Salma and McDermott, 2012 [14], and incubated overnight with at 4°C with primary MEF2D and KLF6 antibodies (1:100) diluted in 1.5% goat serum. Cells were washed three times with PBS for 10 minutes and incubated with the appropriate tetramethyl rhodamine iso-thiocyanate (TRITC)/fluorescein isothiocyanate (FITC)-conjugated secondary antibodies (1:500) in 1.5% goat serum (PBS) for 2 h at room temperature (RT) following 4’,6-diaminidino-2-phenylindole (DAPI) staining for 15 minutes at RT. Cells were washed three times with PBS and cover slips were mounted with DAKO mounting media (Dako) on glass slides. The fluorescence images were captured using Fluoview 300 (Olympus).

### Protein extractions, immunoblotting and reporter gene assays

Cells were harvested using an NP-40 lysis buffer (0.5% NP-40, 50 mM Tris–HCl (pH 8.0), 150 mM NaCl, 10 mM sodium pyrophosphate, 1 mM ethylenediaminetetraacetic acid (EDTA) (pH 8.0), 0.1 M NaF) containing 10 μg/ml leupetin and aprotinin, 5 μg/ml pepstatin A, 0.2 mM phenylmethylsulfonyl fluoride and 0.5 mM sodium orthovanadate. Protein concentrations were determined using the Bradford method (Bio-Rad) with BSA as a standard. We used 20 μg of total protein extracts for immunoblotting, diluted in sample buffer containing 5% β-mercaptoethanol, and boiled. Transcriptional assays were done using Luciferase reporter plasmids. The cells were harvested for these assays using 20 mM Tris, (pH 7.4) and 0.1% Triton-X 100, and the values obtained were normalized to β-galactosidase activity expressed from a constitutive SV40-driven expression vector and represented as relative light units (RLU), or in some cases, corrected Luciferase values for control, reporter alone transfections were arbitrarily set to 1.0, and fold activation values were calculated. Bars represent the mean (n = 3) and error bars represent the standard error of the mean (n = 3).

### Co-immunoprecipitation assays

Protein extracts were prepared as described above. Immunoprecipitation was performed using the ExactaCruz kit (Santa Cruz Biotechnology), as per manufacturer’s instructions. Precipitated proteins were separated by SDS PAGE and immunoblotting of proteins was performed as described above.

### Chromatin immunoprecipitation (ChIP)

ChIP experiments followed the guidelines set by EZ ChIP™ (Upsate) with minor modifications. Approximately 1× 10^7^ C2C12 cells were fixed with 1% formaldehyde (Sigma) for 15 minutes at 37°C. Fixing was quenched by Glycine (Bioshop, Burlington, ON Canada) at a final concentration of 0.125 M. Cells were collected in PBS containing phenylmethylsulfonyl fluoride (PMSF) (Sigma) and protease inhibitor cocktail (Roche, Laval, Quebec, Canada). Cells were collected at 5000 rpm for 5 minutes at 4°C. Cells were lysed using Wash Buffer I (10 mM HEPES pH 6.5, 0.5 M ethylene glycol tetraacetic acid (EGTA), 10 mM EDTA, 0.25% Triton X-100, protease inhibitor cocktail, PMSF) for 5 minutes on ice. Nuclei were collected and resuspended in Wash Buffer II (10 mM HEPES pH 6.5, 0.5 mM EGTA, 1 mM EDTA, 200 mM NaCl, protease inhibitor cocktail, PMSF) for 10 minutes on ice. Nuclei were again collected and then treated with nuclear lysis buffer (50 mM Tris–HCl pH 8.1, 10 mM EDTA, 1% SDS). Chromatin was sheared using a Misonix sonicator to produce 500 bp fragments. Crosslinked sheared chromatin was collected following a 15-minute spin at maximum speed. Twenty percent of total chromatin was set aside as input. Sheared crosslinked chromatin was diluted 1:10 with immunoprecipitation (IP) dilution buffer (0.01% SDS, 1.1% Triton-X 100, 1.2 mM EDTA, 16.7 mM Tris–HCl pH 8.1, 167 mM NaCl) and incubated with antibody overnight at 4°C with rocking. Protein G Dynabeads (Invitrogen) were blocked with 20 μg salmon sperm DNA in IP dilution buffer (15 μl of beads + 135 μl IP dilution buffer + 20 μg salmon sperm DNA per IP) overnight at 4°C with rocking. We incubated 152 μl of pre-blocked beads with the IP reaction at 4°C for 1 h. Dynabead-bound antibody-chromatin complexes were washed using IP Wash Buffer I (20 mM Tris pH 8.1, 2 mM EDTA, 150 mM NaCl, 1% Triton-X 100, 0.1% SDS) and II (20 mM Tris pH 8.1, 2 mM EDTA, 500 mM NaCl, 1% Triton X-100, 0.1% SDS), each incubated for 10 minutes at 4°C, and followed with two washes in Tris-EDTA (TE) buffer at 4°C. Protein-DNA complexes were freed from Dynabeads through the addition of elution buffer (0.1 M NaHCO3, 1% SDS) for 30 minutes at RT. To separate protein from DNA, samples were treated with 12 μl of 5 M NaCl (BioShop) at 65°C for 4 h or overnight. Protein was further degraded by the addition of Proteinase K (Sigma), EDTA, Tris pH 6.5 for 1 h at 45°C. DNA samples were then purified using a PCR clean up kit (Qiagen, Mississauga, ON, Canada).

### Quantitative (q)PCR

ChIP-qPCR analysis of the KLF6 promoter was done using BioRad Sybr Green as per the user manual with a final primer concentration of 0.5 μM. The antibody used in ChIP was 5 μg αMEF2 (sc-313X; Santa Cruz Biotechnology, Inc.). The equivalent amount of rabbit IgG (12–370, Millipore) was used as a control in each ChIP. Sequences of the primers flanking the ME2 site on the KLF6 promoter were: 5’-CTGCAACGTTGGGCTGTA-3’ and 5’-TTGGAAAGACGTCTCACAGG-3’. Each sample was run in triplicate and then analyzed using percent input or fold enrichment.

## Results and discussion

### MEF2D and KLF6 expression and co-localization in the nucleus in skeletal myoblasts

**Figure 1:**
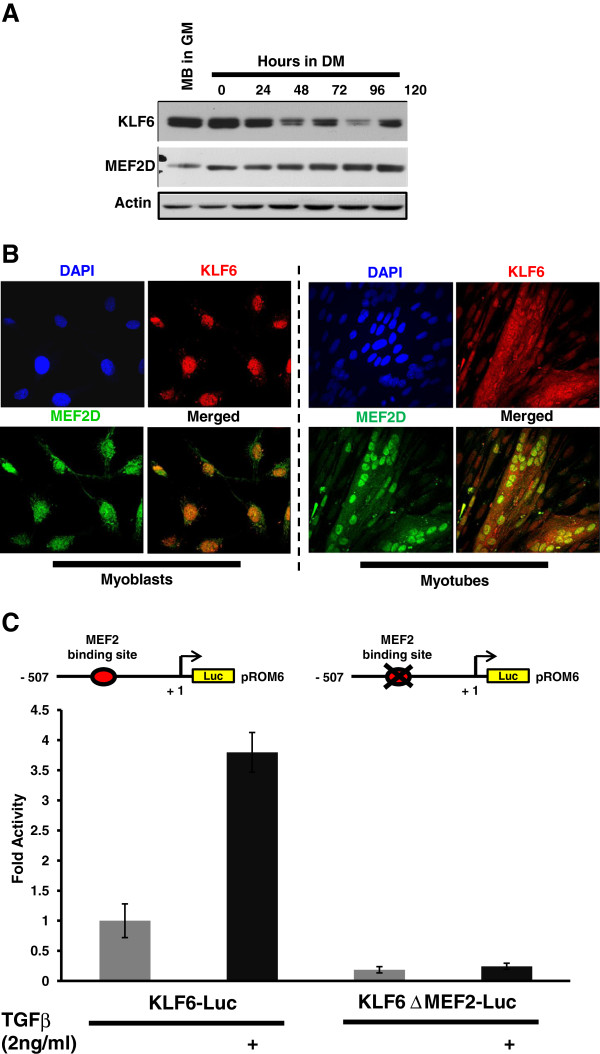
**Western blot analysis reveals that Krüppel-like factor 6** (**KLF6) and Myocyte enhancer factor 2 (MEF2D) are co-expressed in C2C12 myoblasts.** (**a**) Myoblasts were cultured in growth medium (10% serum), followed by serum withdrawal (2%) for 144 h and harvested at 24-h time intervals. Cells were then lysed and equal amounts of protein (20 μg) were used for western blot analysis. The levels of the indicated proteins were assessed by a standard immunoblotting technique using specific primary antibodies for each. Actin was used as a loading control. (**b**) Immunocytochemistry reveals that KLF6 and MEF2D are co-localized in the nucleus at the myoblast stage but to a lesser extent in differentiated myotubes. C2C12 cells were treated as previously described by Salma and McDermott, 2012 [14]. We used 4’,6-diaminidino-2-phenylindole (DAPI) staining for nuclear staining; green and red were used for MEF2D and KLF6 respectively and were then merged. (**c**) Transforming growth factor β (TGFβ) treatment potentiates the KLF6 promoter region through MEF2. KLF6 promoter constructs (pROM6 Luc and pROM6 ΔMEF2 Luc) were used, and Luciferase activities were analyzed upon serum withdrawal, with and without 2 ng/ml TGFβ treatment as indicated.

Since KLF6 was identified in the skeletal muscle transcriptome [15], and has also been shown to be an MEF2D target gene that is involved in the cell survival pathway in primary embryonal hippocampal neurons [14], and since MEF2D is also a crucial regulator of skeletal myogenesis, we wanted to investigate the role of KLF6 in skeletal myoblasts. We determined that KLF6 and MEF2D are indeed both co-expressed in C2C12 myoblasts, and are co-localized in the nucleus using western blot analysis and immunocytochemistry respectively (Figures 1a and 1b). Endogenous expression of KLF6 is detected in C2C12 myoblasts in growth conditions and sustained upon serum withdrawal and throughout the course of myogenic differentiation up to 120 h. Interestingly, we observed that KLF6 protein expression is downregulated at 48 h, upregulated at 72 h, downregulated at 96 h and upregulated again at 120 h in a reproducible manner that is not easily explainable at this point (Figure 1a). Immunofluorescence labeling was conducted to observe the cellular localization of KLF6 with respect to MEF2D in proliferating myoblasts and then in differentiated myotubes. The data indicated strong nuclear localization of both KLF6 (red) and MEF2D (green) in conjunction with nuclear (blue) DAPI staining in myoblasts, and less so in differentiated myotubes (Figure 1b). Since TGFβ has also been shown to regulate KLF6 expression, we tested the effect of TGFβ on previously characterized KLF6 reporter gene constructs (pROM6-Luc and pROM6-Luc ΔMEF2). Serum was withdrawn 24 h after transfection and treatment with 2 ng/ml TGFβ for 24 h was carried out as indicated in the figure. The data illustrates a 4-fold increase in transcriptional activity of pROM6-Luc in response to TGFβ treatment, but no effect on pROM6-Luc ΔMEF2, indicating that TGFβ regulates the KLF6 promoter, which requires that the MEF2 *cis* element is intact (Figure 1c).

### MEF2A/D expression is not required for KLF6 protein expression in skeletal myoblasts

**Figure 2:**
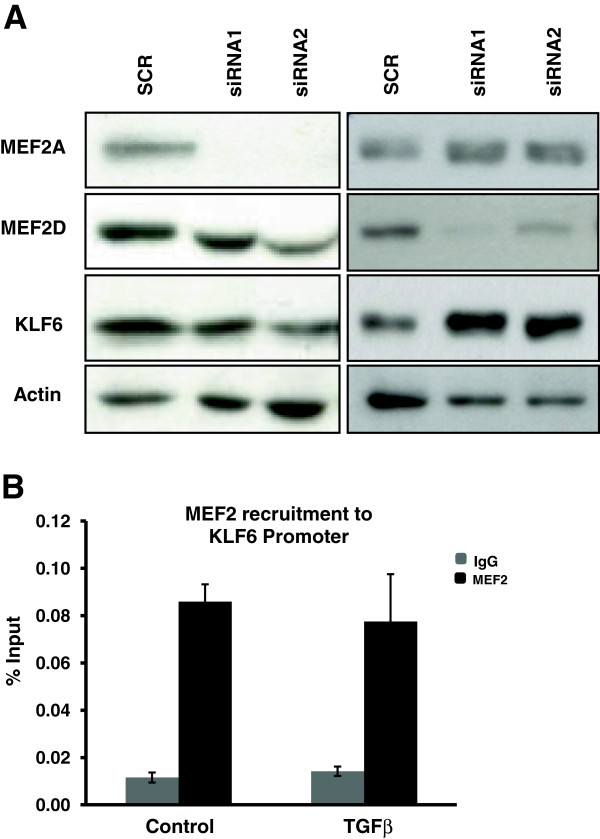
**Myocyte enhancer factor 2 (MEF2)A/D RNA silencing reveals that MEF2A/D expression is not required for endogenous Krüppel-like factor 6 (KLF6) protein expression (a).** In contrast siMEF2D appears to de-repress endogenous KLF6 protein levels. (**b**) Chromatin immunoprecipitation analysis of MEF2 recruitment onto the KLF6 promoter revealed no change upon Transforming growth factor β (TGFβ) treatment.

Since we had already observed that TGFβ regulates the KLF6 promoter through MEF2 we wanted to assess the effect of MEF2A/D knock down using RNA silencing (Figure 2a). Although siRNA2 for MEF2A appears to affect KLF6 expression slightly, this observation did not indicate a strong and consistent effect. On the other hand, siMEF2D appears to de-repress KLF6 expression. Since MEF2D is a potent Histone deacetylase 4 (HDAC4) co-factor, siMEF2D might be preventing the recruitment of HDAC4 to the promoter and hence de-repressing KLF6. Contrary to our initial hypothesis, these data indicate that MEF2 is not necessarily required for KLF6 expression, or that its requirement is only at the myoblast stage when the cells are responsive to TGFβ signaling. To further analyze this observation, we assessed MEF2 recruitment on the KLF6 promoter with or without TGFβ treatment (Figure 2b). These data indicate that while MEF2 is indeed recruited to the KLF6 promoter in C2C12 myoblasts, there is no change in MEF2 recruitment upon TGFβ treatment compared to the control, implicating a different mechanism for TGFβ activation of KLF6.

### TGFβ regulates KLF6 through a Smad3-specific pathway and inhibits skeletal myogenesis through an MEK/ERK-specific pathway

**Figure 3:**
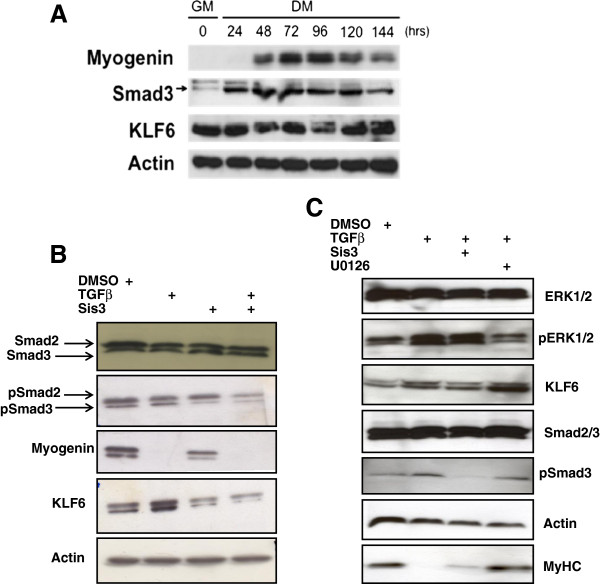
**Western blot analysis revealed that Smad3 and Krüppel-like factor 6 (KLF6) are co-expressed in C2C12 myoblasts (a).** Myogenin was used as a protein marker for differentiation and actin was used as a loading control. Pharmacological manipulation of the Transforming growth factor β (TGFβ) signaling pathway reveals that TGFβ regulates KLF6 protein expression through Smad3 but not MEK/ERK MAPK. (**b**) Western blot analysis indicates that 2 ng/ml TGFβ treatment elevates KLF6 protein expression and that this effect is abrogated in the presence of 5 μM of specific inhibitor of Smad3, Sis3. TGFβ treatment also inhibited the myogenic differentiation marker, myogenin protein expression level, and this effect was not abrogated by Sis3. (**c**) Western blot analysis revealed that TGFβ treatment enhances KLF6 expression through Smad3 but not ERK1/2 MAPK and that TGFβ treatment repressed myogenic differentiation through ERK1/2 MAPK but not Smad3; 10 μM U0126 was used as an inhibitor of the MEK/ERK MAPK pathway, 5 μM Sis3 was used for Smad3 inhibition and 2 ng/ml TGFβ were all used as indicated. Actin was used as a loading control.

Since Smad3 is activated in proliferating myoblasts and is also regulated by TGFβ, we observed that Smad3, along with MEF2 and KLF6, are co-expressed in skeletal myoblasts (Figure 3a). To further investigate the effect of TGFβ on KLF6 we used well-documented pharmacological inhibitors of the Smad and ERK1/2 Mitogen activated protein kinase (MAPK) pathways. We tested the effect of TGFβ on KLF6 protein expression in C2C12 myoblasts in the presence and absence of a Smad3 inhibitor, Sis3 (Figure 3b). The data in Figure 3b reveal that indeed, TGFβ treatment increases KLF6 protein levels and this corresponded with a decrease in myogenin as an indicator of myogenic differentiation. Interestingly, pharmacological inhibition of Smad3 with 5 μM Sis3 reduced TGFβ-induced KLF6 protein expression but had no effect on myogenin. This indicates that TGFβ regulates KLF6 and myogenin through two distinct pathways. Smad2/3 and phospho-Smad2/3 antibodies were used as positive controls for Sis3 treatment since Sis3 inhibits Smad3 phosphorylation and hence its translocation into the nucleus [33]. Since TGFβ also regulates the MEK stands for MAP kinase, ERK kinase Kinase (MEK)/ERK(1/2) MAPK pathway we wanted to test the effect of pharmacological inhibition of that pathway on KLF6 using 10 μM U0126. The data summarized in Figure 3c confirm that TGFβ induces KLF6 protein expression while inhibiting myotube formation (using sarcomeric myosin heavy chain expression as an indicator). In this experiment Smad3 inhibition repressed TGFβ induction of KLF6 but did not reverse the effects on Myosin heavy chain (MyHC) (Figure 3c). Strikingly, pharmacological inhibition of ERK1/2 had no effect on KLF6 levels but instead rescued myotube formation and MyHC expression, thus supporting the idea that TGFβ regulates KLF6 and myogenic differentiation through Smad3 and ERK1/2 distinctively.

### TGFβ induces cell proliferation in C2C12 myoblasts through KLF6

**Figure 4:**
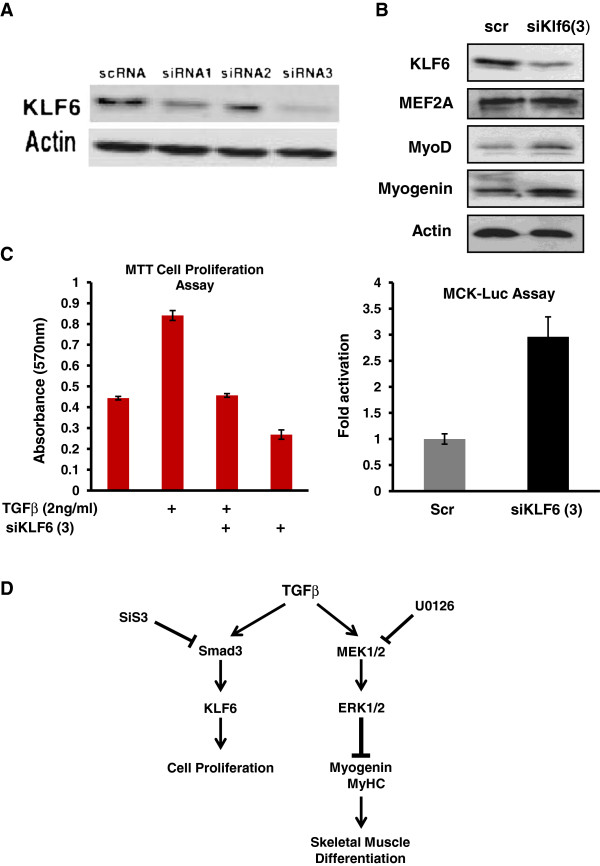
**Krüppel-like factor 6 (KLF6) RNA silencing reveals that KLF6 protein expression was successfully repressed, particularly by siRNA3, which was used in subsequent experiments (a).** (**b**) KLF6 RNA silencing resulted in (i) increased MyoD and myogenin protein levels, (ii) enhanced MCK Luciferase activity and, (iii) reduced transforming growth factor β (TGFβ) induced cell proliferation. (**c**) Cell proliferation was measured using the MTT cell proliferation assay kit. The number of proliferating cells is directly proportional to the absorbance at 570 nm. TGFβ treatment doubled the number of proliferating cells and this effect was repressed with KLF6 silencing. (**d**) A schematic summary of the data presented, in which TGFβ/Extracellular signal regulated kinase (ERK) signaling represses myogenic differentiation while TGFβ/Smad signaling regulates KLF6 gene expression and myoblast proliferation.

Since TGFβ represses skeletal myogenesis by retaining cells in a proliferative state, we wanted to test the effect of KLF6 mRNA silencing using siRNA-mediated gene silencing. siRNA3 was chosen as the most efficient in depleting KLF6 expression as shown in Figure 4a. Subsequent KLF6 silencing resulted in increased MyoD and myogenin protein expression (Figure 4b, upper panel) and this corresponded with a 2.5-fold increase in muscle creatine kinase (MCK) promoter (Figure 4b, lower panel). Furthermore, an MTT cell proliferation assay was performed, and the data showed that at 24 h, 2 ng/ml TGFβ treatment doubles the number of proliferating cells (Figure 4c). This effect is largely negated following KLF6 gene silencing, thus implicating KLF6 in the proliferative response to TGFβ signaling. In support of this, siKLF6 on its own reduced the number of proliferating cells indicating a functional role in proliferation of skeletal myoblasts (Figure 4c).

## Conclusions

In this study we report a novel role for KLF6 in skeletal myoblasts. Based on our data we propose that KLF6 is a downstream effector of the TGFβ/Smad3 pathway that regulates cell proliferation in skeletal myoblasts. We identify Smad3 as a key regulator of KLF6 expression, through TGFβ. In addition we were able to functionally distinguish between the TGFβ/Smad and TGFβ/MAPK pathways in that TGFβ inhibits skeletal myogenesis through the MEK/ERK (1/2) MAPK pathway and concomitantly enhances cell proliferation through Smad3-mediated induction of KLF6 expression. Our findings are summarized in Figure 4d. Many myopathies and muscle loss disorders have been linked with increased TGFβ signaling [34] and hence, our findings identify KLF6 as a potential therapeutic target for such pathological conditions, as well as for cancers, such as embryonal rhabdomyosarcoma, where TGFβ promotes cell proliferation [35].

## Abbreviations

Bp: Base pairs; BSA: Bovine serum albumin; ChIP: Chromatin immunoprecipitation; DAPI: 4’,6-diaminidino-2-phenylindole; DM: Differentiation media; DMEM: Dulbecco’s modified Eagle’s serum; EDTA: Ethylenediaminetetraacetic acid; EGTA: Ethylene glycol tetraacetic acid; EMT: Epithelial-to-mesenchymal transitions; ERK: Extracellular signal regulated kinase; FITC: Fluorescein isothiocyanate; GM: Growth media; HEPES: 4-(2-hydroxyethyl)-1-piperazineethanesulfonic acid; IgG: Immunoglobulin G; IP: Immunoprecipitation; KLF6: Krüppel-like factor 6; Luc: Luciferase; MAPK: Mitogen activated protein kinase; MCK: Muscle creatine kinase; MEF2: Myocyte enhancer factor 2; MEK: MAP Kinase, ERK Kinase Kinase; MRF: Muscle regulatory factor; MyHc: Myosin heavy chain; PBS: Phosphate-buffered saline; PMSF: Phenylmethylsulfonyl fluoride; qPCR: Quantitative polymerase chain reaction; RLU: Relative light units; RT: Room temperature; siRNA: Small interfering RNA; Sis3: Smad3 inhibitor; TE: Tris-EDTA; TGFβ: Transforming growth factor beta; TRITC: Tetramethyl rhodamine iso-thiocyanate; U0126: MEK/ERK inhibitor

## Competing interests

The authors declare that they have no competing interests.

## Authors’ contributions

MGD designed the experiments, performed drug treatments, siKLF6 experiments, and KLF6 functional assays, and drafted the manuscript. JS identified KLF6 as a MEF2D target gene and carried out co-localization and, immunofluorescence experiments. MB performed activity assays and western blotting. SW conducted ChIP analysis and siMEF2 experiments. LZ conducted western blots. JCM conceived of the study, and participated in its design and coordination, and helped to draft the manuscript. All authors read and approved the final manuscript.
